# Signals Getting Crossed in the Entanglement of Redox and Phosphorylation Pathways: Phosphorylation of Peroxiredoxin Proteins Sparks Cell Signaling

**DOI:** 10.3390/antiox8020029

**Published:** 2019-01-23

**Authors:** John J. Skoko, Shireen Attaran, Carola A. Neumann

**Affiliations:** 1 Department of Pharmacology and Chemical Biology, School of Medicine, University of Pittsburgh, Pittsburgh, PA 15261, USA; sha60@pitt.edu; 2 Women’s Cancer Research Center, University of Pittsburgh Medical Center Hillman Cancer Center, Pittsburgh, PA 15213, USA; 3 Magee-Women’s Research Institute, Magee-Women’s Hospital of UPMC, Pittsburgh, PA 15213, USA

**Keywords:** peroxiredoxin, Prdx, Prx, redox signaling, cell signaling, phosphorylation

## Abstract

Reactive oxygen and nitrogen species have cell signaling properties and are involved in a multitude of processes beyond redox homeostasis. The peroxiredoxin (Prdx) proteins are highly sensitive intracellular peroxidases that can coordinate cell signaling via direct reactive species scavenging or by acting as a redox sensor that enables control of binding partner activity. Oxidation of the peroxidatic cysteine residue of Prdx proteins are the classical post-translational modification that has been recognized to modulate downstream signaling cascades, but increasing evidence supports that dynamic changes to phosphorylation of Prdx proteins is also an important determinant in redox signaling. Phosphorylation of Prdx proteins affects three-dimensional structure and function to coordinate cell proliferation, wound healing, cell fate and lipid signaling. The advent of large proteomic datasets has shown that there are many opportunities to understand further how phosphorylation of Prdx proteins fit into intracellular signaling cascades in normal or malignant cells and that more research is necessary. This review summarizes the Prdx family of proteins and details how post-translational modification by kinases and phosphatases controls intracellular signaling.

## 1. Reactive Oxygen and Nitrogen Species in Biology

In biological systems, redox reactions can regulate intracellular levels of reactive oxygen (ROS) and nitrogen species (RNS) to control a multitude of intracellular processes. These reactive species include both radical and non-radical forms of ROS and RNS such as superoxide anion (O_2_^•^^−^), hydrogen peroxide (H_2_O_2_), hydroxyl radical (^•^OH), nitric oxide (NO^•^), nitrogen dioxide (NO_2_^•^) and peroxynitrite (ONOO^−^). The relative reactivity of these species spans several log orders of magnitude to affect the half-life and distance traveled within the intracellular environment. The hydroxyl radical is the most reactive of these species with a half-life on the order of 10^−9^ s, while H_2_O_2_ is much less reactive in comparison with a half-life of 10^−3^ s [1]. Many of these reactive species were initially regarded primarily as harmful oxidants due to the recognition that an overabundance of ROS and RNS cause damage to DNA, proteins, lipids and carbohydrates. ROS and RNS were therefore thought to require reduction to more inert forms in order to maintain intracellular homeostasis and prevent pathophysiological damage. Further research has indicated that ROS and RNS play more complex roles in the cell with mounting evidence supporting a role for these species as mediators at lower concentrations to control protein function and coordinate cell-signaling pathways. 

There are diverse sources of ROS that emerge from several organelles within the cell. The intracellular concentration of H_2_O_2_ has been estimated to be on the order of 1 to 10 nM [2] under basal conditions and reach 0.5 to 0.7 µM during oxidative signaling [3]. Mitochondria have been suggested to be a primary source of ROS due to the byproducts of oxidative phosphorylation to produce ATP. H_2_O_2_ production has been determined to arise from as much as 1–2% of the total oxygen utilized in isolated rat liver mitochondria during respiration [4], but there is debate as to the magnitude [5] and the concentration of H_2_O_2_ produced in vivo [6]. Complex I [7,8] and III [9] produce a large proportion of the ROS generated within the mitochondria as O_2_^•^^−^ [10], which is then reduced to H_2_O_2_ through catalytic dismutation by MnSOD [11]. The steady-state levels of O_2_^•^^−^ have been suggested to be relatively low [6] based on the enzymatic reaction rate (k = 10^9^ M^−1^·s^−1^) [12] and mitochondrial concentrations of MnSOD, which have been measured to be more than 10 µM in isolated rat liver mitochondria [13]. Other organelles that produce ROS include peroxisomes [14], the endoplasmic reticulum [4,15] and lysosomes [16]. The plasma membrane and cytoplasm also produce ROS through the action of NADPH oxidase [17], prostaglandin synthase [18], lipoxygenases [19] and xanthine oxidase [20] and transition metals [21], respectively. All of these sources contribute to both the intracellular H_2_O_2_ load as well as the extracellular secretion of H_2_O_2_, which can reach 2 µM in stimulated neutrophils [22]. 

Reactive species have a complex relationship in cancer. An overabundance of reactive species can cause cancer initiation and drive progression and accumulation of additional DNA mutations to support further growth (reviewed in References [23,24]). Antioxidants can, therefore, serve a protective role to block reactive species from initiating the carcinogenic process. Suppression of reactive species does not always impart a less malignant phenotype though. Research has shown that increasing the intracellular concentrations of antioxidants, and antioxidant enzymes in cancer cells can similarly drive cancer progression and more invasive characteristics [25]. Reactive species and the endogenous signaling pathways that aid to scavenge them have been suggested to follow a U-shaped curve where a surplus or deficiency of either can increase the risk of cancer [24,26]. Intracellular enzymatic reactive species scavengers are therefore necessary to carefully balance the processes of proliferation and survival against induction of cell death to maintain homeostasis and guard against malignancy. 

## 2. Enzymatic H_2_O_2_ Scavengers

H_2_O_2_ is a particularly important reactive molecule with second messenger function. Spatiotemporal features of the molecule enable it to possess reactivity, yet move through biological macromolecular microenvironments intracellularly within different organelle compartments and intercellularly to neighboring cells. Organ and cellular systems coordinate the balance of the H_2_O_2_ through enzymatic-catalyzed reductant proteins in conjunction with the pro-oxidant sources mentioned above to form an interconnected network that maintains homeostasis or drives oxidative signaling. The enzymatic metabolism of H_2_O_2_ is primarily catalyzed through the action of catalase, glutathione peroxidases (GPx) and peroxiredoxins (Prdx). While all three enzymatically metabolize H_2_O_2_, important biochemical and biological differences exist. Insight into the basal function of the three enzymes in vivo can be drawn from the phenotypic effects observed in gene knockout studies. Deletion of catalase displays no overt pathological changes under basal conditions in mice, while GPx1 knockout mice were found to be smaller sized [27] and harbor a predisposition to the development of cataracts [28,29,30]. These changes contrast with the more extreme pathophysiological changes that exist upon deletion of Prdx1 and 2. Prdx1 knockout mice exhibit increased oxidative damage to DNA and cancer incidence at various sites throughout the animal as well as shortened lifespan and hemolytic anemia [31]. Deletion of the yeast Prdx homolog tsa1 has also been shown to have deleterious effects such as increased oxidative damage, thermosensitivity, mutagenesis and genomic instability [32,33]. Prdx2 knockout mice were similarly found to develop hemolytic anemia but do not show an increased incidence of cancer [34].

## 3. Catalase and GPx

Catalase is localized within peroxisomes and catalyzes the decomposition of H_2_O_2_ via an iron heme porphyrin complex [35]. A two-step mechanism is utilized where H_2_O_2_ is enzymatically scavenged through oxidation followed by reduction of the active site heme iron. The active site heme is initially oxidized to an oxyferryl species, which is subsequently reduced by a second H_2_O_2_ molecule to regenerate enzymatic function. Sequestration of catalase to a single organelle enables control of peroxisomal H_2_O_2_ levels but also requires H_2_O_2_ derived from other intra- and extracellular sources to diffuse to the peroxisome in order for catalase-dependent catalysis to occur. Catalase exhibits a high H_2_O_2_ turnover rate [36], but sequestration in combination with other enzyme kinetic properties, such as a K_m_ close to 100 mM in human erythrocytes [36], yield an enzyme that is less effective when H_2_O_2_ concentrations are low. Contrasting catalase, GPx family members are found within several organelles. There are eight family members in the glutathione peroxidases (GPx) family (GPx1-8). GPx1-4 and 6 utilize a selenocysteine active site and glutathione (GSH) as a co-factor to reduce H_2_O_2_ [37,38]_._ GPx1 and 4 are present in most tissues, with GPx1 expression found in the cytoplasm and mitochondria, while the phospholipid hydroperoxide reducing GPx4 is found in the plasma membrane and cytoplasm [38]. Although GPx1 and 4 do not display true Michaelis Menten kinetics, the second-order rate constant of the two-part catalytic cycle is in the range of 10^5^ M^−1^ s^−1^ [38]. Gpx5 and GPx7-8 are non-selenoproteins that possess an active site cysteine residue, which harbors decreased peroxidase activity in contrast to the other selenocysteine-containing family members [39]. These proteins have been suggested to play a redox sensor role.

## 4. Prdx

The Prdx family has six members (Prdx1-6) that are present in many cellular compartments. Prdx1, 2 and 6 are located in the cytoplasm, and nucleus; Prdx3 is localized to the mitochondria; Prdx4 is found in the endoplasmic reticulum; and Prdx5 is located in the peroxisomes, cytoplasm and mitochondria [40]. The Prdx family have 1 or 2-cysteine (Cys)-dependent reaction mechanisms to reduce H_2_O_2_ to water. The family is subdivided into groups classified as 2-Cys (Prdx1-4), atypical 2-Cys (Prdx5) and 1-Cys (Prdx6) isoforms based on their structure and mechanism of action [41]. The active site peroxidatic Cys (Cys_p_) is conserved among all family members at roughly 50 amino acids from the N-terminus. The Cys_p_ is highly reactive to H_2_O_2_ due to its surrounding amino acids, which yield rate constants on the order of 10^6^ to 10^8^ M^−1^ s^−1^ [42]. Typical 2-Cys are susceptible to overoxidation of the Cys_p_ under conditions of heightened H_2_O_2_ concentrations, resulting in a cysteine sulfinic acid, which can be further oxidized to a Cys sulfonic acid to cause irreversible inactivation of the enzyme [43,44]. Typical 2-Cys Prdx proteins are predisposed to overoxidation due to the presence of the C-terminal YF motif, which associates in a loop motif with a GGLG bordering the Cys_p_ [41,44,45]. These structural elements can participate in the overoxidation of the catalytic cysteine; however, these motifs are not present in mammalian Prdx5 and Prdx6, suggesting these isoforms are more resistant to H_2_O_2_ induced sulfinylation reactions that are characteristically observed in typical 2-Cys Prdxs [40,45,46].

### 4.1. 2-Cys Typical Prdx

**Figure 1:**
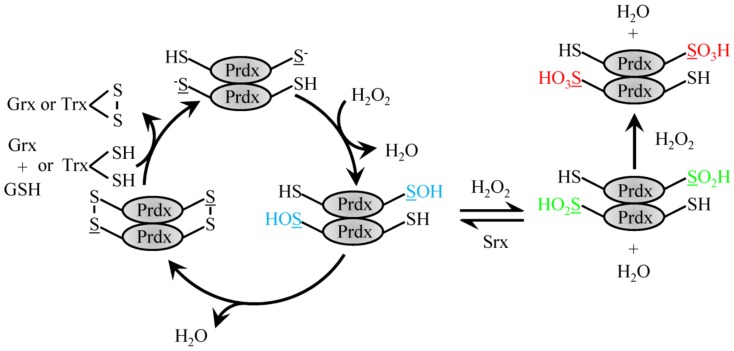
Reduction of H_2_O_2_ by typical 2-Cys Prdx. H_2_O_2_ recycling by 2-Cys typical Prdx family members involves the conversion of the peroxidatic Cys to sulfenic acid. Sulfenylated Prdx protein can then either form a disulfide bond with the resolving Cys on the dimeric partner protein to be recycled via Trx or GSH and Grx1 or be further oxidized to the sulfinic acid form, which can be reversed with Srx or fully over-oxidized sulfonic acid form.

Homodimerization of Prdx proteins in a an N-terminus head to C-terminus tail fashion enables 2-Cys family members to align the Cys_p_ to the mechanistically important resolving Cys located on the opposing Prdx homodimerization partner near the C-terminus. Prdx1 and 2 homodimers can associate non-covalently to form larger decameric complexes that are ordered as a pentamer of dimers to form a doughnut-like structure [44,46]. In 2-Cys Prdx, the Cys_p_ is oxidized by H_2_O_2_ to a sulfenic acid moiety, which then forms a disulfide bond with the resolving Cys on the homodimerization partner [46,47] (Figure 1). The disulfide bound dimer destabilizes decameric Prdx to cause dissociation of the complex [48,49]. The redox reaction cycle can be regenerated by reducing the formed homodimer disulfide bond with Trx [50]. The Cys_p_ can become overwhelmed in the presence of high levels of H_2_O_2_ and become overoxidized to form Cys sulfinic or further sulfonic moieties that lack peroxidase activity. The rate constant of the sulfenic acid form of Prdx2 with H_2_O_2_ to form the sulfinic Prdx is on the order of 10^4^ M^−1^ s^−1^ [51] The sulfinic form was found to be reversible via enzymatic reduction by sulfiredoxin protein [52]. In addition to the classic Trx recycling, a second redox cycle has recently been described for Prdx2. The sulfenic Cys_p_ can be adducted with GSH (rate constant 500 M^−1^ s^−1^) under physiological concentrations to protect from overoxidation and recycle with Grx1 [53]. 

### 4.2. 2-Cys Atypical Prdx

**Figure 2:**
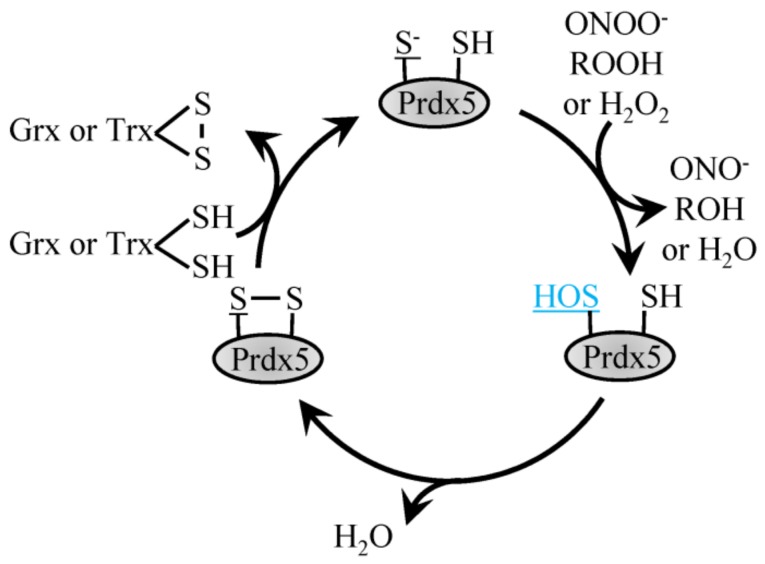
Reduction of H_2_O_2_ by atypical Prdx5. H_2_O_2_, ROOH and ONOO^-^ recycling by 2-Cys atypical Prdx5 involves the conversion of the peroxidatic Cys to sulfenic acid, which then forms a disulfide bond with the resolving Cys to be recycled by Trx or Grx1.

The atypical Prdx5 follows a similar reaction mechanism but contrasts typical 2-Cys Prdx by forming an intramolecular disulfide bond with the internal resolving Cys as opposed to an intermolecular disulfide bond (Figure 2) [45,54]. Characterized by its amino acid sequence, Prdx5 differs most when compared to the other Prdx family members, with roughly only 30% sequence identity. In comparison to other family members, Prdx5 has a broad cellular localization. Prdx5 can be found in the mitochondria, peroxisomes, cytoplasm and nucleus [45]. Both prokaryotic and eukaryotic peroxiredoxins function to catalyze the reduction of H_2_O_2_ and ONOO^-^ with varying enzymatic efficiencies. Of all the Prdx family members, human Prdx5 was the first mammalian Prdx shown to react with ONOO^-^ with a rate constant in the range of 10^7^ M^−1^ s^−1^. In comparison, catalytic reduction of H_2_O_2_ by Prdx5 occurs slower, with a rate constant of roughly 10^5^ M^−1^ s^−1^. These different enzymatic activities suggest that when Prdx5 is localized to cellular compartments where other Prdx family members are also present, Prdx5 may more specifically reduce ONOO^-^ or lipid peroxides as opposed to H_2_O_2_ [45].

### 4.3. 1-Cys Prdx

**Figure 3:**
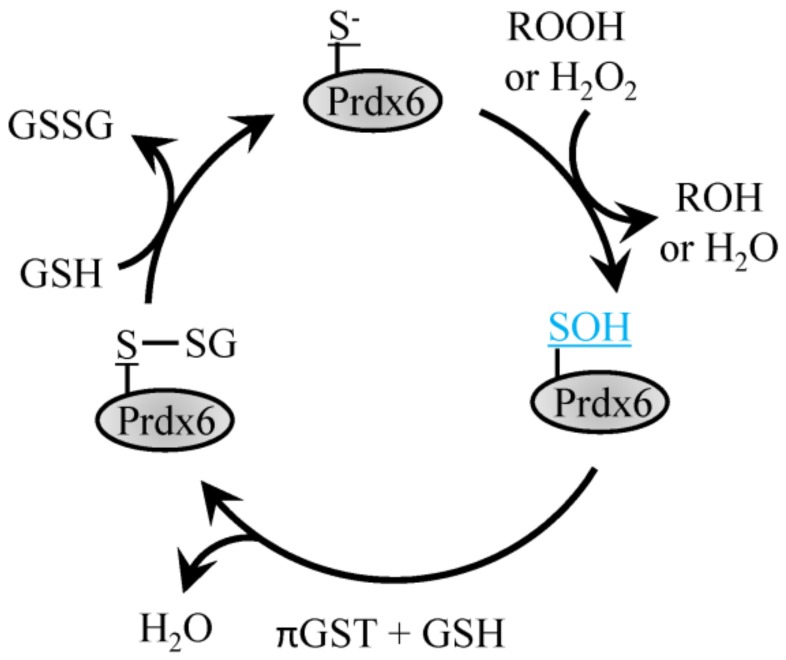
Reduction of H_2_O_2_ by Prdx6. H_2_O_2_ or ROOH recycling by 1-Cys Prdx6 involves the conversion of the peroxidatic Cys to sulfenic acid, which then forms a disulfide bond with GSH through the action of πGST and can be recycled back to reduced Prdx6 with another GSH.

The 1-Cys Prdx6 protein forms homodimers but does not form a disulfide bond following oxidation of the Cys_p_ and instead exists in the sulfenic acid form that is reduced with GSH (Figure 3) [55]. Prdx6 is a 1-Cys peroxiredoxin which can function as both, glutathione peroxidase and phospholipase A_2_ [55]. Prdx6 differs from its other family members in that it does not utilize thioredoxin as an electron donor and can function as a Se-independent peroxide. Prdx6 has two primary functions, the first, as a peroxidase functioning to reduce oxidized phospholipids utilizing amino acids Cys47, Arg132 and His39 [55]. Enzymatic activity is also present at a second site that enables Ca^2+^-independent phospholipase A2 activity with the aid of Ser32, Glu140 and His26. 

## 5. The Role of Prdx in Signaling through Redox Relay

The peroxidase activity of 2-Cys Prdx has been tied to redox sensor functions to control cell signaling pathways through protein coupling reactions [56]. Prdx2 has recently been shown to participate in a thiol-disulfide exchange reaction with the transcription factor STAT3 to repress transcriptional activation [57]. The highly sensitive Cys_p_ of Prdx2, therefore, acts akin to an oxidative receptor that transfers the oxidative signaling equivalents to a partnering target protein through a Cys redox relay. This mechanism enables the coordination of oxidative signaling to target proteins in the absence of high concentrations of H_2_O_2_ or highly reactive Cys elements in target proteins. Redox relays also exist within the cytoplasm for Prdx1 [58] and have been further investigated in larger scale studies. CRISPR-Cas9 deletion of the cytoplasmic Prdx family members Prdx1 and 2 in HAP1 cells found that cells without Prdx1 or 2 had less oxidation of cytoplasmic protein thiols globally and further support the importance of the redox relay hypothesis [59]. Other cellular compartments also show redox relay actions such as in the ER for Prdx4 [60] and Gpx7 [61]. This mechanism is currently under further exploration to determine its role in redox regulation of other previously labeled “redox active” proteins that possess rate constants on the order of 10 to 10^2^ M^−1^ s^−1^ [62] but reside outside close proximity to an H_2_O_2_ generating source and within a cellular environment with abundant highly reactive peroxidases. 

## 6. Phosphorylation of Prdx to Promote Proliferation and Survival

Outside of oxidation of the Cys_p_ in Prdx proteins, other factors have been described to alter peroxidase activity and structure including local microenvironmental aspects such as pH [63], ionic strength [64] and temperature [65]. Expression of Prdx family members in various cancers has been shown to be altered in a number of studies (reviewed in Reference [66]), but total protein expression is only one component to control functional signals within the intracellular environment. Post-translational modifications can affect peroxidase activity, structure and coordinate cell signaling through changes in Prdx activity via phosphorylation at different sites within the protein. Phosphorylation of Prdx proteins to modulate structure and function has been established to coordinate pro-survival and death pathways by modulating intracellular H_2_O_2_ levels within intracellular compartments temporally. Heightened local production of H_2_O_2_ is an important step during cell proliferation in normal and malignant cells. The reversible oxidation and inactivation of protein tyrosine phosphatases (PTPs) enable protein tyrosine kinases (PTKs) to phosphorylate downstream targets without PTP-dependent removal of the phosphate moiety. PTPs are more readily targeted by H_2_O_2_ due to the enhanced reactivity of the low pK_a_ active site cysteine residue, which causes increased susceptibility to oxidation compared to PTKs. Cell signaling cascades, therefore, utilize local H_2_O_2_ to coordinate signal transduction pathways to promote growth [67,68]. To develop pools of H_2_O_2_ within different intracellular compartments, cells require not only the production of H_2_O_2_ but the inactivation of peroxidases to drive phenotypic responses. As Prdx proteins are highly sensitive and enzymatically responsive to low levels of H_2_O_2_, the cell has developed pathways to inhibit peroxidase activity within the cell during cell proliferation. Suppression of Prdx1 activity by phosphorylation has been shown to play a role during the cell cycle in two different intracellular compartments, namely at the plasma membrane during growth factor signaling and within the nucleus during the transition to mitosis.

### 6.1. Protein Tyrosine Kinase Phosphorylation of Prdx1 at the Plasma Membrane

A classic counter-example to the redox relay cell signaling coordination by Prdx described above is provided by phosphorylation of Tyr194 on Prdx1 by Src [69]. Local inactivation of Prdx1 peroxidase activity generates increased zonal concentrations of H_2_O_2_ through the action of Nox [70] at the plasma membrane, which is required to drive growth factor receptor tyrosine kinase signaling [71,72]. Of importance to highlight, various growth factor receptors, Src family kinases, and NOX enzymes are localized to the plasma membrane specifically at lipid rafts. Many studies to date have suggested that H_2_O_2_ production by NOX1 is not sufficient alone to promote and sustain signaling [44,69]. Rather, inactivation of Prdx1 by phosphorylation on Tyr194 causes localized and sustained accumulation of H_2_O_2,_ thereby allowing target proteins such as the Src family kinases to become oxidized [69]. To sustain further phosphorylation and inactivation of Prdx1, local accumulation of H_2_O_2_ near lipid rafts is necessary to both simultaneously activate Src family kinases and inactivate PTPs. H_2_O_2_-mediated inhibition of PTPs is therefore crucial for growth factor signaling as activation of PTKs alone is not adequate to promote sufficient levels of protein tyrosine phosphorylation [69].

Phosphorylation on Tyr194 in response to treatment of cells with EGF or PDGF displayed isoform selectivity for Prdx1 in comparison to Prdx2 [69]. Studies showed that siRNA knockdown or pharmacological inhibition of Src reduced the phosphorylation. The Cys_p_ was found to be protected from overoxidation in phosphorylated protein during co-treatment of cells with growth factor and H_2_O_2_. In vitro studies of the phosphorylated protein found the dimeric form of the Prdx1 was present without decamers. In vivo wound healing experiments exhibited a peak in Tyr194 phosphorylation after 1 day, which remained for 1 week.

### 6.2. Cdk1-Cyclin B Phosphorylation of Prdx1 in the Nucleus

Local H_2_O_2_ accumulation through inactivation of Prdx1 by phosphorylation is also important in the nucleus during mitosis [73]. During early mitosis, Prdx1 bound to the centrosome is inactivated by Cdk1-cyclin B phosphorylation of Prdx1 at Thr90 to promote inactivation of the dual-specificity PTP Cdc14B by elevated H_2_O_2_ [74]. Inactivation of Cdc14B and possibly other mitotic exit phosphatases sensitive to inactivation by H_2_O_2_ enables active Cdk1-cyclin B to transition cells to late mitosis where Prdx1 can be dephosphorylated to promote deactivation of Cdk1 via dephosphorylation by the now activated Cdc14B. The importance of the centrosomal H_2_O_2_ during the transition to mitosis was evaluated by expressing catalase fused to a centrosomal targeting sequence, which inhibited entry into mitosis. 

### 6.3. Phosphorylation of Prdx1 to Enhance Peroxidase Activity

While the studies above indicate post-translational modifications that serve to reduce peroxidase activity, not all Prdx phosphorylation is repressive to enzymatic activity. The T-cell-originated protein kinase (TOPK) can phosphorylate Prdx1 on Ser32 to enhance peroxidase activity [75]. Investigation of proteins that bind TOPK in response to ultraviolet light B irradiation of RPMI7951 cells by mass spectrometry identified that Prdx1 phosphorylation reduced accumulation of H_2_O_2_ in vitro and ex vivo. In the absence of TOPK via siRNA decrease, cells were more sensitive to UVB-induced apoptosis. Melanoma cells expressing wild-type but not Ser32Ala Prdx protein was more resistant to UVB irradiation. Whether Prdx2 can also be phosphorylated by TOPK is unknown but may not be seen in experiments with RPMI7951 cells, which only express high levels of Prdx1. 

### 6.4. Prdx6 Phosphorylation to Control Phospholipase A_2_ Activity

Phosphorylation of Prdx6 has been found to attenuate phospholipase A_2_ (PLA_2_) activity, which is unique to the Prdx family. As Prdx6 phosphorylation increases PLA_2_ activity, phosphorylation of the protein may mirror protein overexpression seen in bladder [76], liver [77] and lung [78,79,80] cancer. Within secretory organelles and lysosomes, Prdx6 has been shown to function in lung surfactant metabolism via aiPLA_2_ activity [81]. Acidic calcium-independent PLA_2_ (aiPLA_2_) functions to degrade surfactant phospholipids and aide DPPC (1-palmitoyl-2-[3H] 9,10-palmitoyl-sn-glycero-3-phosphorylcholine) synthesis through the deacylation-reacylation pathway, by providing the required substrate, lyso-PC. Phosphorylation of Prdx6 provides an important functional role as a PLA_2_; phosphorylated Prdx6 maintains activity at both acidic and cytosolic pH [81]. Treatment of isolated rat alveolar type II cells with phorbol myristic acid (PMA) was found to increase Prdx6 phosphorylation and aiPLA_2_ activity through the MAPK pathway [81]. ERK1, ERK2, p38α, p38β2, p38γ and p38δ but not JNK, were able to phosphorylate Prdx6 in vitro and mass spectrometry analysis revealed ERK2 specifically phosphorylated Prdx6 at Thr177. Purified Thr177Glu Prdx6 protein exhibited 2-fold higher PLA_2_ activity compared to Thr177Ala protein. 

Phosphorylation of Prdx6 on Thr177 was found to be critical for activation of NOX2 in pulmonary microvascular endothelial cells (PMVEC) by angiotensin II (Ang II) or PMA [82]. Phosphorylation of Prdx6 caused cytoplasmic to plasma membrane translocation and *Prdx6^−/−^* PMVEC displayed dramatically decreased plasma membrane translocation of the NOX2 components Rac1 or p47*^phox^* following Ang II stimulation. Upon treatment with the known MAPK inhibitor, U0126, Prdx6 phosphorylation, PLA_2_ activity and ROS were significantly decreased. This result suggested that MAPK activation mediates phosphorylation of Prdx6 resulting in its translocation to the cellular membrane, whereby, PLA_2_ activity can function to promote NOX2 activation [82]. Whether peroxidase activity is altered in phosphorylated Thr177 is unclear. Wu et al. suggested that peroxidase activity was not affected in Thr177Ala Prdx6, but Chhunchha et al. have found there is a 25% reduction in peroxidase activity [81,83]. Chhunchha et al. further determined that peroxidase-deficient C47S Prdx6 protein had 30% decreased PLA_2_ activity when compared to wild-type protein [83].

## 7. Phosphorylation of Prdx to Induce Cell Death 

In addition to phosphorylation pathways that promote cell proliferation and pro-survival by increasing H_2_O_2_ in the cell, phosphorylation of Prdx proteins has been tied to cell death. Interestingly, Prdx1 phosphorylation at Thr90, which aids in driving the cell cycle through mitosis in the nucleus as described above, can also be targeted by tumor suppressor proteins. The difference between activation of survival or death pathways based on phosphorylation of the same protein at the same amino acid highlights the importance of location and timing of Prdx phosphorylation and the possible involvement of additional phosphorylation of Prdx1 at Thr183 to control activity. The following sections will detail the mechanistic studies that implicate Prdx phosphorylation with death. While some examples below highlight neurodegenerative pathways as examples of inactivation by phosphorylation, further cancer-focused work needs to be undertaken to extend these mechanisms as both Cdk5 and LRRK2 activity have been implicated in cancer. Cdk5 activity has been noted to be involved in proliferation, migration, invasion, metastasis, the epithelial to mesenchymal transition, the DNA damage response and angiogenesis in many forms of human cancer (as reviewed in Reference [84]). The LRRK2 p.G2019S activating mutation has also been associated with an increased risk of cancer [85,86]. 

### 7.1. Phosphorylation of Prdx1 by Mst1

Mammalian sterile 20–like kinase-1 (Mst1) and Mst2 have been shown to suppress tumor formation in the liver and intestines in vivo [87,88,89,90]. Mst1 and Prdx1 have been shown to regulate the activity of one another. Cells treated with H_2_O_2_ cause Prdx1 to bind Mst1, which was found to promote Mst1 activation and enhance apoptosis [91]. Loss of Prdx1 was further shown to be important for Mst1 stimulation by H_2_O_2_. In contrast, phosphorylation of the Thr90 residue of Prdx1 by Mst1 and possibly Mst2 has been described to inactivate peroxidase activity [92]. Full-length Mst1 is localized in the cytoplasm, but caspase cleavage causes nuclear translocation of the kinase [93]. Whether Mst1 inactivation of Prdx1 is cell compartment-dependent or specific to the nucleus or cytoplasm is unknown. Mst1 can additionally phosphorylate Thr183 in the C-terminus of the Prdx1, which may also inactivate peroxidase activity as indicated by in vitro assays with site-directed mutagenesis engineered Thr183Asp Prdx1 purified protein [92]. Expression of Thr183Asp Prdx1 protein in *Prdx1^−/−^* mouse embryonic fibroblasts (MEFs) showed heightened levels of the DNA damage biomarker phosphorylated Ser139 H2AX following treatment with H_2_O_2_. This finding suggests that the inactivation of Prdx1 by Mst1 could potentially cause a positive feedback loop whereby excess H_2_O_2_ further activates Mst1. That inactivation of Prdx1 by phosphorylation can promote Mst1 activation, while knockdown of Prdx1 can inhibit Mst1 activity, suggests that a more complex pattern of regulation is present that may involve both oxidation and phosphorylation.

### 7.2. Prdx2 Inactivation by Phosphorylation by Cdk5 Complexes

Prdx2 phosphorylation has been primarily investigated in pathological brain diseases and injury. Mitochondrial dysfunction and excessive oxidative stress are believed to be critical facilitators of the pathogenesis of Parkinson’s disease (PD). Neurons have oxidative metabolism systems in place, such as Prdxs, to manage and prevent the accumulation of deleterious levels of oxidative stress but can be overwhelmed when ROS scavengers are decreased or inactivated. In the context of PD, Prdx2 associates with a Cdk5 kinase complex via p35 or the proteolytically cleaved products of p35, p10 and the hyper-activated p25 [94]. Cdk5/p35 and Cdk5/p25 complexes were shown to phosphorylate Prdx2 at Thr89, resulting in a decrease in Prdx2 peroxidase activity and neuronal death in MPP^+^ treated cells in vitro and MPTP treated mice in vivo. Furthermore, Cdk5 activation was shown to mediate dopaminergic (DAergic) neurodegeneration by downregulation of Prdx2 peroxidase activity which ultimately leads to significant elevations of intracellular ROS and DAergic neuron death [94]. This observation was extended to human PD where it was demonstrated that phosphorylated Prdx2 was elevated in neurons in the soma of clinical PD patient samples [94]. Another report identified that phosphorylation of Prdx2 by the Cdk5/p25 complex could be inhibited by expression of p10 in SH-SY5Y cells and MPP+ treated rat cortical neurons [95]. Expression of p10 was further found to reduce intracellular ROS in these cells. As Prdx2 was originally identified to interact with p35 via precipitation of the p10 fragment [94], p35 seems to have a bifunctional regulatory mechanism to regulate Prdx2-dependent cell death through phosphorylation. The ability of Cdk5 to phosphorylate Prdx proteins was also shown to include both Thr89 Prdx2 and Thr90 Prdx1 in Alzheimer’s disease models [96]. Cdk5-dependent phosphorylation of Prdx protein caused mitochondrial damage due to oxidative stress induced by Aβ or glutamate.

The Cdk5-Prdx2 pathway has also been shown to cause neuronal death in ischemia models [97]. Glutamate excitotoxicity in primary cerebellar granule neurons was identified to cause increased Thr89 phosphorylation in Prdx2 in wild-type but not *p35^−/−^* neurons. Glutamate-induced neuronal cell death was rescued by viral expression of wild-type or Thr89Ala Prdx2 but not Thr89Glu Prdx2. Ischemia-dependent neuronal cell death generated by 4-vessel occlusion in hippocampal CA1 neurons could also be rescued by expression of wild-type Prdx2 but not Thr89Glu in vivo [97]. Phosphorylation of Prdx2 exhibited reliance on Cdk5 activity as dominant negative Cdk5 could block phosphorylation.

### 7.3. Prdx3 Phosphorylation in the Mitochondria by LRRK2

Prdx3 is expressed in mitochondria and has been shown to be important for maintaining mitochondria mass and membrane potential in Myc-transformed fibroblasts and breast cancer cells [98]. The debilitating effects of phosphorylation-induced repression of Prdx3 peroxidase activity have been explored in the area of neurodegeneration. Phosphorylation of Thr146 in Prdx3 by leucine-rich repeat kinase 2 (LRRK2) protein was associated with decreased peroxidase activity by measuring NADPH oxidation of isolated Prdx3 [99]. The importance of the peroxidase activity of Prdx3 in the mitochondria was demonstrated with knockdown of Prdx3 in neuronal SPx3 cells containing activated LRRK2, which led to increased mitochondrial cytochrome c release and caspase 3 levels. These results were shown to be relevant in postmortem brain tissue samples from patients diagnosed with Parkinson’s disease harboring a common p.G2019S activating mutation (rs34637584:A>G) in LRRK2, which similarly displayed heightened levels of Prdx3. This mechanism was further explored in a *Drosophila* model that expressed activated kinase mutant LRRK2, where overexpression of Prdx3 rescued flies from decreased lifespan, depletion of dopaminergic neurons and dysfunctional mitochondria in flight muscles of flies [100]. The importance of the scavenging activity of Prdx3 was shown by pharmacologically rescuing the dysfunctional phenotypic effects of activated kinase mutant LRRK2 in *Drosophila* with the Prdx mimetic ebselen, which diminishes H_2_O_2_ and peroxynitrite. 

## 8. Dephosphorylation of Prdx Family Members

Reports detailing the importance of interactions between kinases and Prdx family members suggest dephosphorylation may represent an equally significant process. The absence of a Prdx phosphate removal mechanism would require phosphorylated Prdx protein to be degraded or sequestered for future coordination of various signaling pathways. Protein recycling through proteasomal degradation may occur, but this would represent an energy-intensive process requiring *de novo* expression of unmodified protein during signaling events. While information regarding the dephosphorylation of Prdx family members is relatively scarce, some information is available. The protein phosphatase PP2A has been identified to dephosphorylate Prdx1 when phosphorylated at Thr90, which could be blocked with okadaic acid [101]. Dephosphorylation of Prdx1 was regulated by the peptidylprolyl cis/trans isomerase Pin1 and found to be attenuated in *Pin1^−/−^* MEFs. The association between Pin1 and Prdx1 was enhanced in the presence of 50 μM H_2_O_2_ and could be inhibited by pharmacologically blocking Cdk1 activity or mutation of Thr90 Prdx1 to Ala. Further experiments found that Pin1 could also bind Prdx2, 3 and 4 but not in cell lysates containing Thr-Pro motif mutants Thr89Ala Prdx2, Thr146Ala Prdx3 and Thr162Ala Prdx4. 

Dephosphorylation of Prdx4 has also been demonstrated by Ptp1b [102]. Granulocyte colony-stimulating factor (G-Csf) stimulation of Ptp1b-proficient MEFs increased phosphorylation of Prdx4 over 2 h as measured by immunoblot using phosphor-Tyr antibodies from Prdx4 immunoprecipitated samples. *Ptp1b^−/−^* MEFs exhibited significantly higher levels of Prdx4 phosphorylation in the presence or absence of G-Csf stimulation when compared to Ptp1b-proficient MEFs. It is unclear which Tyr was phosphorylated in *Ptp1b^−/−^* MEFs or the functional consequences, but the authors suggested that the Tyr phosphorylation may decrease Prdx4 peroxidase activity. Overall, the existence of phosphatases that dephosphorylate Prdx family members is an area that requires further exploration. 

## 9. Unexplored Regulatory Prdx Phosphorylation

The current state of knowledge of phosphorylation as a means to modulate Prdx function has been relatively understudied. While biochemical details exist for modification at Ser32, Thr90, Thr183 and Tyr194 and attempts have been made to evaluate Thr18, the consequences of phosphorylation of Prdx1 at multiple sites are unclear. Phosphorylation of Prdx1 Thr90 and Thr183 would be expected to perform similar functions as both sites are targeted by Mst1, decrease peroxidase activity and promote the formation of decameric complexes, but not all combinations are as straightforward. There are questions regarding what occurs when contrasting sites are phosphorylated that cause distinct changes to the oligomeric tertiary structure or peroxidase activity. Phosphorylation of Ser32 has been shown to enhance peroxidase activity, but whether this can exert a dominant effect to rescue the decreased peroxidase functionality of Prdx phosphorylated at Thr90, Thr183 or Tyr194 is unknown. The general structure of a phosphorylated Thr90 or Thr183 Prdx1 decamer may also collapse following Tyr194 phosphorylation, which has been shown to lead to homodimers of Prdx1 primarily. Multi-site phosphorylation may be precluded from occurring in some cases due to changes in the three-dimensional protein structure that prevent sequential kinase binding from occurring, but many other proteins have been shown to have multi-layered signal transduction cascades of regulation. The existence of positive and negative phosphorylation feedback loops to control post-translationally modified proteins temporally has been well documented in transcription factors, kinases and other enzymes. 

In our current era where large resource experimental datasets have become more readily available due to technical advancements, large proteomic datasets can be mined to determine if protein modifications are present under a myriad of conditions. Published datasets have emerged that support existence of novel phosphorylated forms of Prdx family members. As modifications of plants have been well described in a previous review by Liebthal et al. [103], we will instead focus on mammalian phosphorylated Prdx residues that are conserved in humans and indicated in the PhosphoSitePlus database. 17 separate phosphorylated species have been identified using high-throughput proteomic techniques for Prdx1 and so far only four of the sites have been biochemically characterized (Table 1). Residues in bold were identified in proteomic screens in cancer cells and may serve cancer-specific functions. Prdx1 represents the most well-characterized member of the Prdx family. As detailed above, Prdx1 can play an important role in coupling phosphorylation-based cell signaling pathways to thiol redox-based signaling systems to control an array of processes including receptor tyrosine signaling and cell division.

Phosphorylation of other members of the Prdx family has been less well described. There is evidence that the cdc2-cyclin B complex is capable of phosphorylating Prdx1, 2, 3 and 4 in vitro but cell compartmentalization concerns were suggested by the authors to preclude Prdx3 and 4 from the likelihood of interacting with Cdk proteins [123]. While Cdk-dependent phosphorylation of Prdx has been more comprehensively detailed for Prdx1, evidence of Prdx2 targeting by Cdk5/p35 and Cdk5/p25 complexes was described to reduce peroxidase activity and cause neuronal death in a toxin-induced model of Parkinson’s disease [94]. Alignment of the protein sequence of Prdx1 and Prdx2 shows a high degree of similarity between the two proteins with 77% identical residues and overlap of several prospective Prdx2 phosphorylation sites, namely, Thr18, Ser31, Thr89, Tyr115, Thr182, Tyr193 and Ser195. As characterization has been performed for the Prdx1 homologs Ser32, Thr90, Thr183, and Tyr194, it is possible that phosphorylation of Prdx2 at these sites similarly alters structure and function as seen for Thr89 in Prdx2 and Thr90 in Prdx1. It is unknown whether these sites have divergent kinetics, kinase specificity or intracellular compartmental or phenotypic effects. In total, 13 phosphorylation sites that have been identified in Prdx2 in proteomic studies and only Thr89 has been characterized (Table 2). 

**Figure 4:**
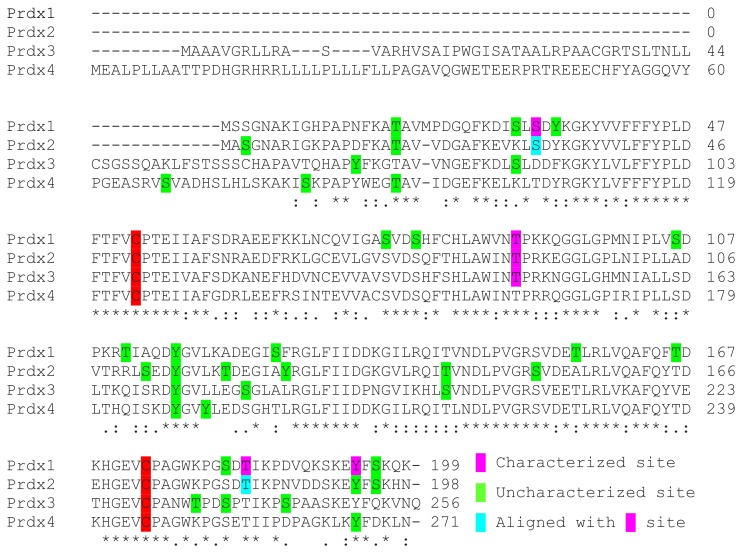
2-Cys Prdx alignment of observed phosphorylation sites. Alignment of the 2-Cys Prdx family members. Phosphorylation sites that have been characterized are highlighted in pink, uncharacterized sites that have been identified in proteomic studies are highlighted in green and sites that are aligned with characterized phosphorylation sites are highlighted in blue.

In comparison to Prdx1 and 2, the number of phosphorylation sites present in large datasets is less abundant for the rest of the protein family. Delineation of the effects of phosphorylation of Prdx3 for Thr146 in LRRK2 kinase mutant cells and flies has been described and suggests phosphorylation is a mechanism utilized to alter peroxidase activity, but other sites have yet to be studied [98,100]. Thr146 of Prdx3 aligns with Thr90 and Thr89 of Prdx1 and Prdx2, respectively, which both have also been characterized to reduce peroxidase activity and may be expected to additionally promote the formation of larger oligomeric complexes upon phosphorylation (Figure 4). Prdx3 phosphorylation has been observed at eight sites, but interestingly, Thr146 has not yet been observed in large proteomic screens (Table 3). General exploration of the effects of phosphorylation on Prdx4 and 5 is lacking and studies have yet to characterize the peroxidase activity or oligomeric structural results of this modification. Proteomic data is the only resource that describes the intracellular phosphorylation of these proteins (Table 4 and Table 5). The number of phosphorylation sites for these proteins is six and five for Prdx4 and 5, respectively. Phosphorylation of Prdx5 on Thr97 or Ser101 of the full-length unprocessed protein (Thr45 and Ser49 of the processed form) is likely to influence peroxidase activity as these residues surround the peroxidatic Cys48 residue. Phosphorylation of the final member of the Prdx family, Prdx6, has been described on Thr177 to elevate its aiPLA_2_ activity but peroxidase specific phosphorylation sites have not yet been detailed. Six phosphorylation sites of Prdx6 have been described in large proteomic datasets (Table 6). Tyr89 is the only Tyr residue present in Prdx6 that was detected to be phosphorylated. Two-dimensional electrophoresis followed by mass spectrometry conducted on Bax inhibitor-1 (*bi-1)^+/+^* and *bi-1^−/−^* mice found that Tyr phosphorylation was increased in *bi-1^−/−^* liver, brain, heart, lung and kidney tissues [145]. The identity and functional consequences of the Tyr phosphorylation site or sites within Prdx6 that were elevated in the knockout mice were not determined, but Tyr89 is potentially the site of interest. Tyr89 Prdx6 may also be phosphorylated in the dorsal striatum of midkine (Mk) knockout mice in cocaine conditioned place preference experiments [146].

## 10. Conclusions and Future Directions

The highly sensitive peroxidatic cysteine thiol of Prdx proteins makes them well suited to sense and control cell signaling due to minor changes in H_2_O_2_, ROOH and ONOO^−^ concentration within multiple cellular organelles. Research efforts have therefore primarily focused on how oxidation of the peroxidatic Cys perturbs cell signaling. While there are many important unrealized details and properties of Prdx oxidation that remain, the effects of other forms of post-translational modifications to Prdx proteins represent another exciting area of exploration. Dynamic changes to oxidation, phosphorylation and other forms of post-translation modifications can change three-dimensional structure and function of Prdx proteins to coordinate the various processes outlined above. In addition, control of Prdx through phosphorylation further integrates redox and signaling cascades. Inactivating phosphorylations of Prdx proteins likely prevent Prdx redox relay reactions as the peroxidatic Cys loses peroxidase activity. Prdx phosphorylation cascades would thus enable attenuation of Prdx-dependent oxidative equivalent transfer to partner proteins and inhibit redox signaling. The role of peroxidase activating phosphorylation sites such as Ser32 of Prdx1 on redox relays is also unknown but may perhaps oppose peroxidase inactivating phosphorylation sites.

Post-translational modifications of Prdx family members have been identified to play a significant role in several signaling cascades, but there remains to be a complete understanding of the function of several putative sites in many family members. The introduction of phosphomimetic and non-phosphorylatable mutants via genomic editing through systems such as CRISPR/Cas9 would be useful to determine the functional consequences of Prdx phosphorylation in normal non-transformed cells as well as cancer cells. Candidate phosphorylation sites to be studied further could be identified from proteomic screens in cancer cells such as those indicated in bold in Table 1, Table 2, Table 3, Table 4, Table 5 and Table 6 above. Several sites have been found in multiple proteomic screens for Prdx1 (Ser32, Ser126 and Tyr194), Prdx2 (Ser112) and Prdx6 (Thr44) and more than half have yet to be characterized. Biochemical evaluations on putative phosphorylation sites that have already been performed have shown changes to phosphorylation sites that can increase or decrease peroxidase activity, in the case of Prdx1 Ser32 or Prdx1 Tyr194, respectively. Prdx peroxidase activity may, therefore, play an intricate role in cancer that could be dependent on the oncogenic driver, cell type or cancer stage. Functional studies in these systems may yield valuable information that helps to better understand the role of phosphorylation in Prdx biology and cancer. Extensions into in vivo models would further develop phosphorylation of Prdx from a systems biology perspective.

## Figures and Tables

**Table 1:** Phosphorylation of Prdx1 can increase or decrease peroxidase activity to coordinate cell signaling. Phosphorylation sites that correspond to human Prdx1 in biochemical assays and proteomic studies are referenced are indicated. Phosphorylation sites that have been identified in proteomic screens in cancer models are in bold.

Prdx1 Residue	Effect	Biochemical Assay References	Proteomic Dataset References
**Thr18**	Function unknown	[92]	[104,105]
Ser30	Function unknown		[106,107,108,109,110,111]
**Ser32**	Enhanced peroxidase activity	[75]	[106,107,108,109,110,111,112,113,114,115,116,117,118,119,120]
Tyr34	Function unknown		[118,121]
Ser77	Function unknown		[122]
Ser80	Function unknown		[122]
Thr90	Decreased peroxidase activity	[73,74,92,96,123]	[107]
Ser106	Function unknown		[105,124]
**Thr111**	Function unknown	[92]	[104,105]
**Tyr116**	Function unknown		[105,118,125]
**Ser126**	Function unknown		[104,119,126]
Thr156	Function unknown	[92]	
Thr166	Function unknown		[105]
**Ser181**	Function unknown		[107,119,127,128,129]
Thr183	Decreased peroxidase activity	[92]	[107,127,130]
**Tyr194**	Decreased peroxidase activity	[69]	[131,132,133,134,135,136]
**Ser196**	Function unknown		[116]

**Table 2:** Phosphorylation of Prdx2 can decrease peroxidase activity to coordinate cell signaling. Phosphorylation sites that correspond to human Prdx2 in biochemical assays and proteomic studies are referenced are indicated. Phosphorylation sites that have been identified in proteomic screens in cancer models are in bold.

Prdx2 Residue	Effect	Biochemical Assay References	Proteomic Dataset References
Ser3	Function unknown		[137]
Thr18	Function unknown		[105]
**Ser31**	Function unknown		[111,115,116,119]
Thr89	Decreased peroxidase activity	[94,95,96,97]	[138]
**Ser112**	Function unknown		[105,115,116,119,126,139,140]
Tyr115	Function unknown		[118,141]
**Thr120**	Function unknown		[111,119,140]
Tyr126	Function unknown		[140]
Thr142	Function unknown		[105,142,143]
**Ser151**	Function unknown		[104,111]
Thr182	Function unknown		[115]
**Tyr193**	Function unknown		[133,134,135,136,144]
Ser195	Function unknown		[144]

**Table 3:** Phosphorylation of Prdx3 can decrease peroxidase activity to coordinate cell signaling. Phosphorylation sites that correspond to human Prdx3 in biochemical assays and proteomic studies are referenced are indicated. Phosphorylation sites that have been identified in proteomic screens in cancer models are in bold.

Prdx3 Residue	Effect	Biochemical Assay References	Proteomic Dataset References
**Tyr71**	Function unknown		[125]
**Ser86**	Function unknown		[105,119]
Thr146	Decreased peroxidase activity	[99,100]	
Tyr172	Function unknown		[118]
**Ser179**	Function unknown		[105,119]
**Ser199**	Function unknown		[104,127]
Thr234	Function unknown		[107,115]
**Ser237**	Function unknown		[107,119,147]
Ser243	Function unknown		[110,147]

**Table 4:** Phosphorylation sites that correspond to human Prdx4 in proteomic studies are referenced are indicated.

Prdx4 Residue	Effect	Proteomic Dataset References
Ser68	Function unknown	[115]
Ser82	Function unknown	[148]
Thr91	Function unknown	[148]
Tyr188	Function unknown	[118]
Tyr191	Function unknown	[118]
Tyr266	Function unknown	[149]

**Table 5:** Phosphorylation sites that correspond to human Prdx5 in proteomic studies are referenced are indicated. Phosphorylation sites that have been identified in proteomic screens in cancer models are in bold.

Prdx5 Residue	Effect	Proteomic Dataset References
**Thr97**	Function unknown	[105,107,116,119,150,151]
**Ser101**	Function unknown	[105,119]
Ser168	Function unknown	[108,112]
Ser171	Function unknown	[111,150]
**Ser182**	Function unknown	[105,112,116,119]

**Table 6:** Phosphorylation of Prdx6 can increase calcium-independent phospholipase A_2_ activity to coordinate cell signaling. Phosphorylation sites that correspond to human Prdx6 in biochemical assays and proteomic studies are referenced are indicated. Phosphorylation sites that have been identified in proteomic screens in cancer models are in bold.

Prdx6 Residue	Effect	Biochemical Assay References	Proteomic Dataset References
Ser32	Function unknown		[111]
**Thr44**	Function unknown		[105,114,116,119,126,128,140,152,153,154,155,156,157]
**Tyr89**	Function unknown		[107,111,131]
**Ser146**	Function unknown		[104,105]
**Thr177**	Enhanced acidic calcium-independent phospholipase A_2_ activity	[81,82,83]	[115,118,153,156]
Ser186	Function unknown		[105,110,154]
